# Role of melatonin in the angiogenesis potential; highlights on the cardiovascular disease

**DOI:** 10.1186/s12950-021-00269-5

**Published:** 2021-02-02

**Authors:** Afshin Rahbarghazi, Marefat Siahkouhian, Reza Rahbarghazi, Mahdi Ahmadi, Lotfali Bolboli, Rana Keyhanmanesh, Mahdi Mahdipour, Hadi Rajabi

**Affiliations:** 1 Department of Physical Education and Sports Sciences, Faculty of Educational Science & Psychology, University of Mohaghegh Ardabili, Daneshgah Street, Ardabil, Iran; 2 Tuberculosis and Lung Disease Research Center, Tabriz University of Medical Sciences, Tabriz, Iran; 3 Drug Applied Research Center, Tabriz University of Medical Sciences, Tabriz, Iran; 4 Department of Applied Cell Sciences, Faculty of Advanced Medical Sciences, Tabriz University of Medical Sciences, Tabriz, Iran; 5 Stem Cell Research Center, Tabriz University of Medical Sciences, Tabriz, Iran

**Keywords:** Melatonin, Angiogenesis, Cardiovascular disease, Regeneration

## Abstract

Melatonin possesses multi-organ and pleiotropic effects with potency to control angiogenesis at both molecular and cellular levels. To date, many efforts have been made to control and regulate the dynamic of angiogenesis modulators in a different milieu. The term angiogenesis or neovascularization refers to the development of *de novo* vascular buds from the pre-existing blood vessels. This phenomenon is tightly dependent on the balance between the pro- and anti-angiogenesis factors which alters the functional behavior of vascular cells. The promotion of angiogenesis is thought to be an effective strategy to accelerate the healing process of ischemic changes such as infarcted myocardium. Of note, most of the previous studies have focused on the anti-angiogenesis capacity of melatonin in the tumor niche. To the best of our knowledge, few experiments highlighted the melatonin angiogenesis potential and specific regulatory mechanisms in the cardiovascular system. Here, we aimed to summarize some previous experiments related to the application of melatonin in cardiovascular diseases such as ischemic injury and hypertension by focusing on the regulatory mechanisms.

## Introduction

Angiogenesis is an essential process in the formation of *de novo* blood vessels from the pre-existing network during physiological and pathological conditions [1]. There are numerous pro- and anti-angiogenesis factors that participate in the dynamics of vascular regeneration. In this regard, a wide array of signaling effectors such as NO, EGF, SDF-1α, TGF)-β 1 and 2, VEGF, etc. participate in the modulation of angiogenesis in pathological and physiological conditions [2–5]. The equilibrium between angiogenesis stimulators and inhibitors directs angiogenic fate in a specific organ [1, 6]. In cardiac tissue, coronary angiogenesis in the basis of blood supply to different parts of the heart [7]. Most of the angiogenic effectors are activated during vessel formation in the embryos and are induced again after ischemic conditions within the cardiac tissue [7]. MI is touted as the most common cause of human death worldwide correlated with partial or complete occlusion of microvasculature in the affected areas [8]. In most MI patients, the lack of sufficient blood supplementation to the infarcted area leads to extensive necrosis and aberrant ECM remodeling [9]. Restorative compensatory mechanisms and promotion of fibroblasts in MI patients contribute to the bulk secretion of type I collagen in the interstitial spaces, leading to insufficient myocardial tissue and diastolic dysfunction [10]. To circumvent these conditions, restoring blood supply and the promotion of angiogenesis in the infarcted area could significantly reduce cardiac remodeling and dysfunction [8]. Angiogenic strategies in MI and cardiovascular disease use valuable approaches by stimulating the formation of *de novo* blood vessels from the pre-existing vascular network [11]. To this end, multiple growth factors have been used in the stimulation of angiogenesis in the ischemic areas and some of these factors such as VEGF and bFGF have reached human clinical trials. The use of these factors did no last longer in clinical medicine due to short half-time, rapid diffusion, poor stability, and uncontrolled angiogenesis rate [12]. Commensurate with these descriptions, it is mandatory to examine and use other endogenous factors with low-side effects and long-term activities in patients with different cardiovascular dysfunctions.

During past decades, the pleiotropic roles of MT have been discovered in different organs. MT is a lipophilic compound and could pass all circulatory system barriers and plays a fundamental role in the function of multiple cell types [6]. Up to now, most experiments have focused on the anti-angiogenic potential of MT in cancer biology [13]. Emerging data revealed that MT is a smart hormone and could act depending on the condition. It seems that MT can suppress or trigger angiogenesis through different signaling pathways leading to diverse biological outcomes [14, 15]. Here, we aimed to summarize some previous experiments related to the application of MT in cardiovascular diseases such as ischemic injury and hypertension by focusing on regulatory mechanisms.

## Biology of MT and mechanism of action

**Fig. 1:**
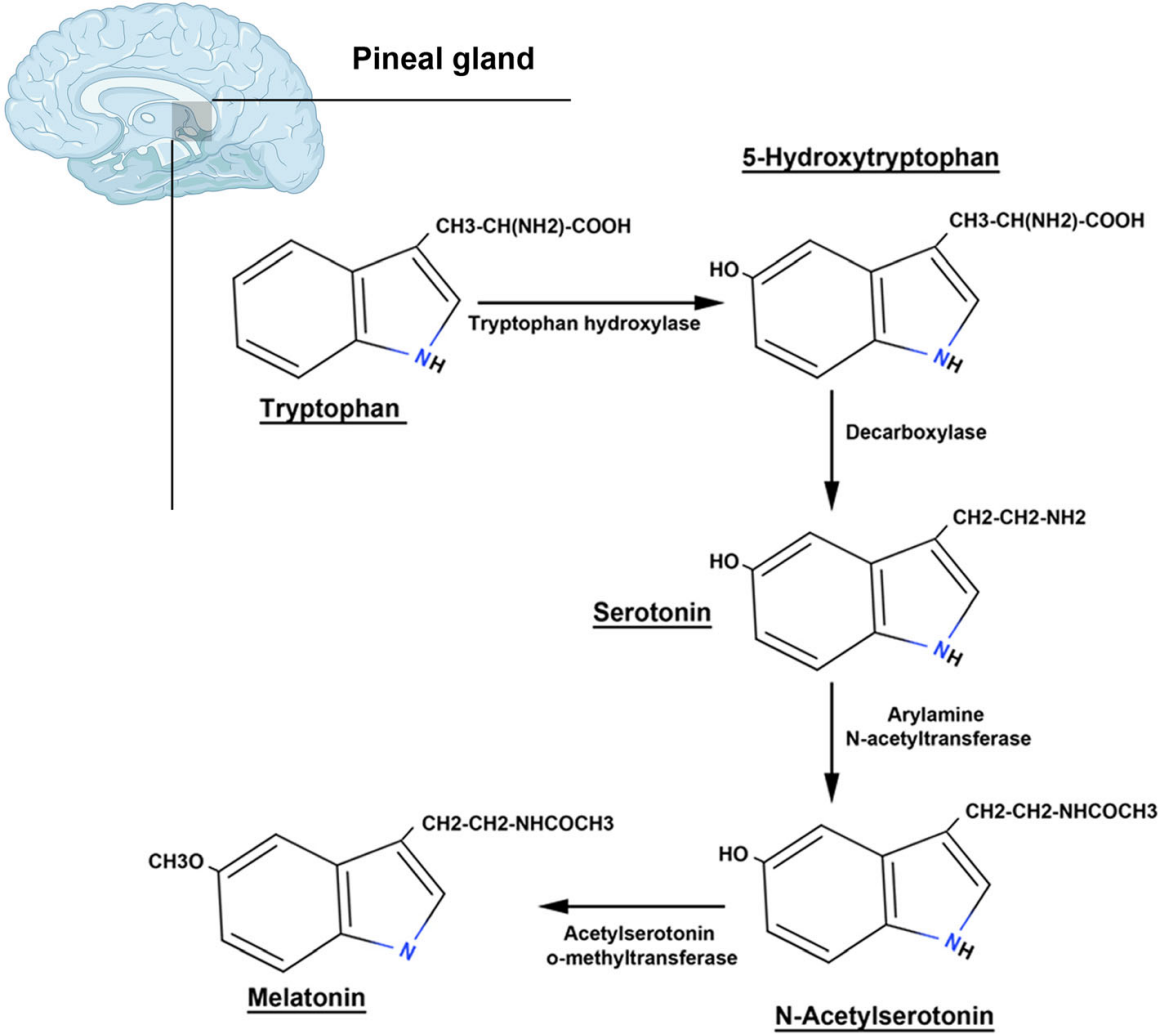
The synthesis of melatonin by the pinealocytes located at the pineal gland. Tryptophan is the precursor of serotonin and melatonin. The conversion of Tryptophan to melatonin occurs via several enzymatic reactions

**Fig. 2:**
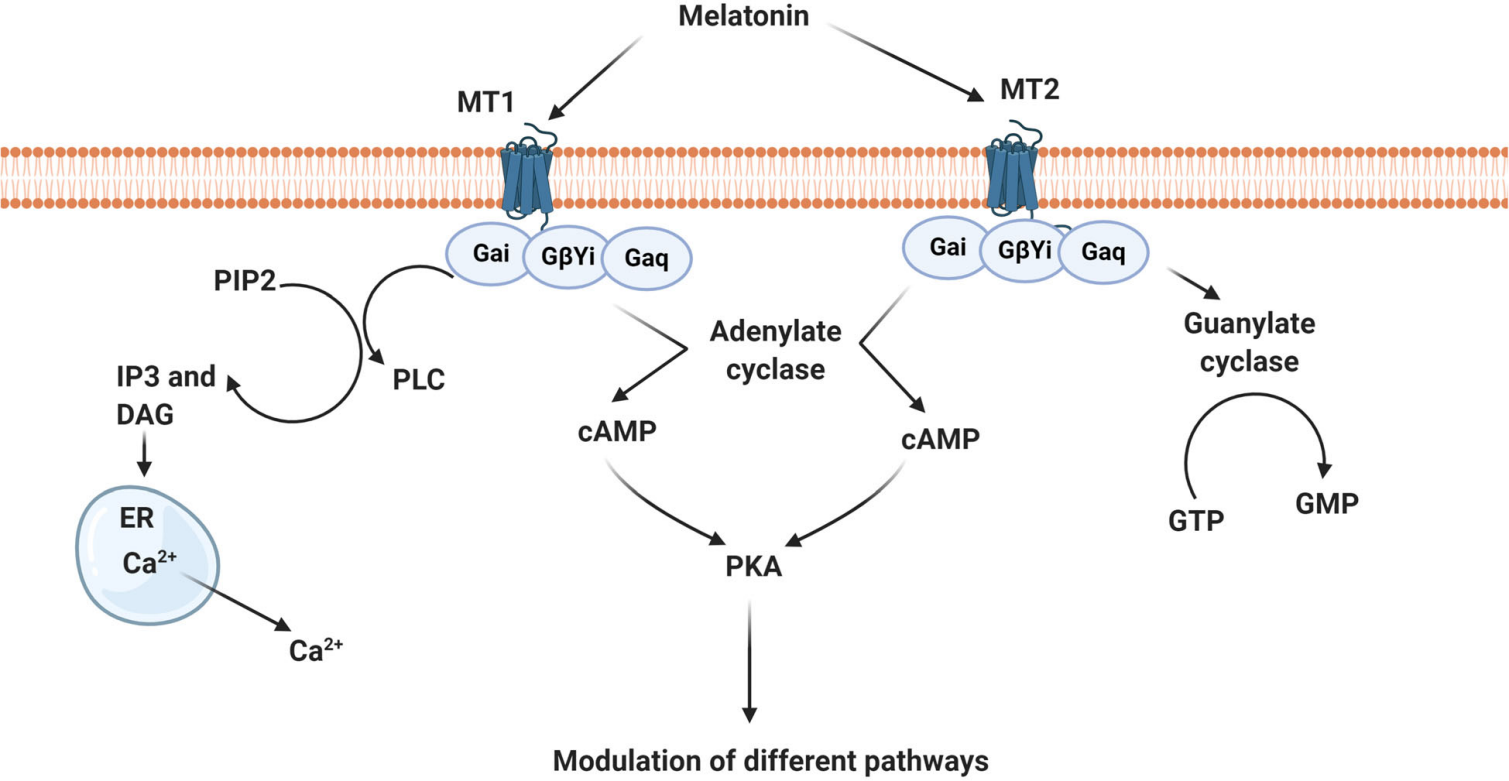
Schematic representation of the melatonin function on the target cells via membrane-bound receptors and different effectors

MT is touted as a pleiotropic hormone with the ability to alter different cellular functions [14]. This hormone with a chemical structure of N-acetyl-5-methoxytryptamine is produced from the amino acid tryptophan in the pituitary gland. First, tryptophan enters inside the active pineal cells, namely pinealocytes, and is converted into the hormone serotonin through a 5-hydroxyindole pathway [16, 17] (Fig. 1). By the activity of tryptophan hydroxylase, tryptophan is converted into 5-hydroxytryptophan. The reaction continues by the aromatic L-amino acid decarboxylase which further converts 5-hydroxytryptophan into 5-hydroxytryptamine which is known as also serotonin. After two consequential biochemical reactions, acetylation and methylation, serotonin is converted into MT. The acetylation and methylation occur through the activity of two distinct enzymes termed serotonin-N-acetyltransferase and hydroxyindole-O-methyltransferase, respectively [18] (Fig. 1). Due to the high metabolic rate and low storage of MT by the pineal gland, its plasma level reflects pineal activity [19]. The systemic level of MT is tightly regulated by the suprachiasmatic nuclei circadian pacemaker and exposure to light [20]. The concentration of MT remains constant during the day and peaks in the darkness. After mid-darkness, MT level increases shortly and reaches a short-term peak, and then diminishes before the onset of light to return to daily levels [21, 22]. It is thought that the production of MT is influenced by different factors such as age, sex, season, and some diseases and decreases with age. Of course, levels of the MT hormone in elderly women are higher than that of older men, and its production in humans is higher in winter compared to the summer [23]. Multiple actions of MT are done by cell membrane-bound receptors and nuclear sites of orphan members of the RZR/ROR super-nuclear receptor family. In this regard, three types of MT receptors have been suggested [24] (Fig. 2). Many physiological functions of MT are done through the physical interaction with membrane G-protein-coupled receptors termed MT1 (linked to Gαi and Gαq subunits) and MT2 (linked to Gαi subunit). MT binds with a high affinity to either MT1 or 2 receptors [25, 26] (Fig. 2). Upon activation of these receptors after exposure to the MT, both receptors are inhibitory to the formation of stimulated cyclic AMP. Alternatively, MT2 also stimulates phosphokinase C activity. In addition to MT1 and MT2 receptors, it has been shown that mammals harbor an intra-cytoplasmic MT3 receptor, a quinone reductase 2A, with a low-affinity binding site for MT [27]. The MT3 receptor, MT3 is less known in humans and is expressed commonly in the liver and kidneys. It seems that this type of MT receptor has a detoxification role and is less abundant in tissues such as the heart, fat, and brain.

**Fig. 3:**
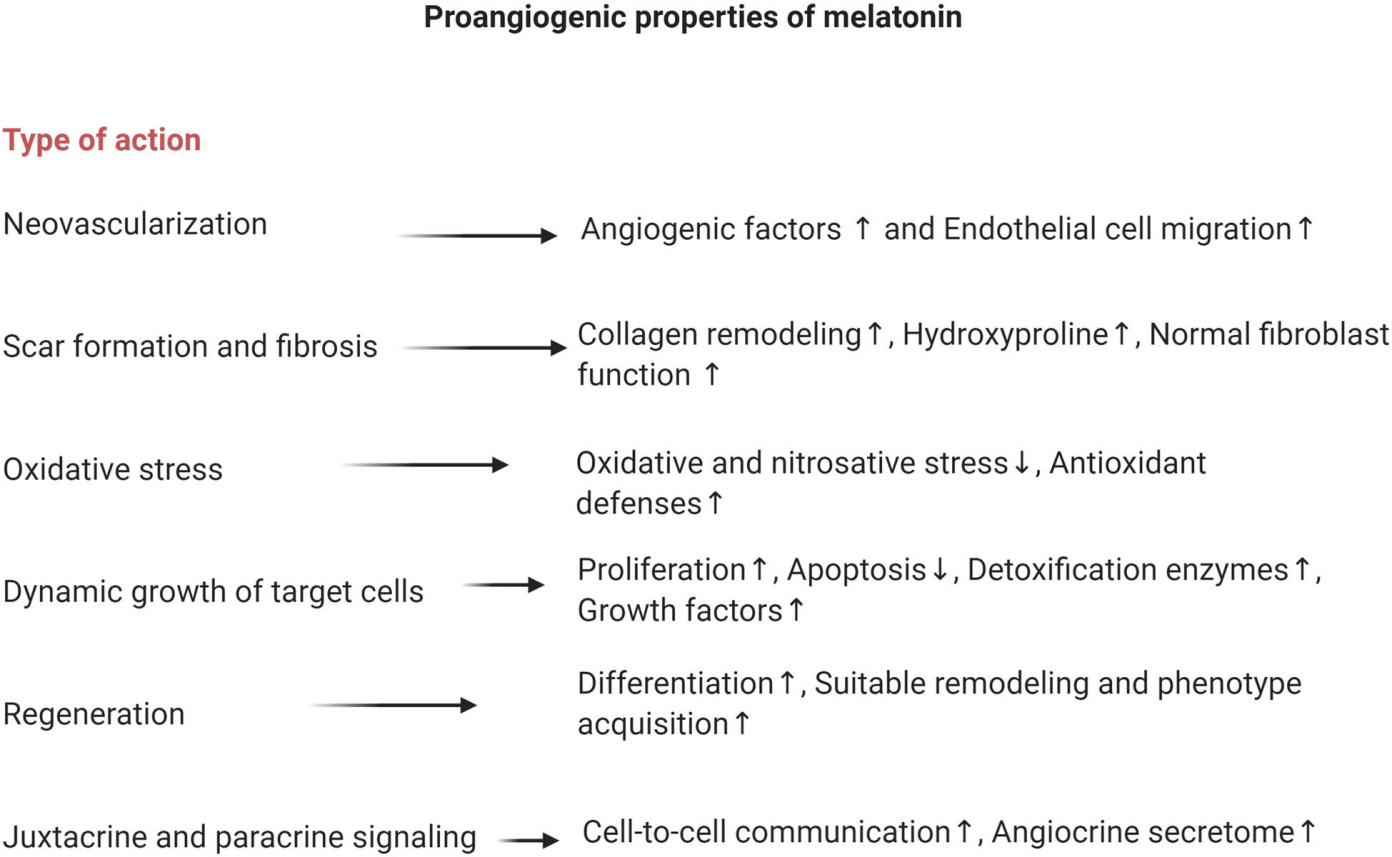
List of different mechanisms in the angiogenic potential of melatonin

Recent data examining MT in *in vivo* and *in vitro* conditions reported that MT could act as a potent antioxidant agent and converts free radicals to harmless final products [28]. ROS is produced under various physiological and pathological conditions, including inflammation and ischemia. Several studies have noted that the transfer of free harmful electrons and hydrogen atoms is the way by which MT could exert an antioxidant activity [29]. MT can have a direct role to stimulates the bioactivity of antioxidant enzymes including SOD, GPx, GR, and Cat while inhibits the activity of lipoxygenase. Commensurate with these descriptions, MT antagonizes, scavenges noxious free radicals, and protects the cells against oxidative damage by increasing the microsomal membrane integrity, and reduction of hydrolases leakage into the cytosol [30]. These events lead to MT-mediated protection and homeostasis of mitochondria function and cell membrane integrity against salient free-radical production. Notably, it was well established that MT potently is stronger compared to the other scavengers like vitamin E, glutathione, and mannitol to neutralize free radicals caused by oxidation procedure and metabolism of unsaturated fatty acids [31].

Although significant progress has been achieved regarding the high potency of MT in preventing, reducing, and reversing different diseases and pathological changes, there is still much attempt to be done. This review article tries to address the potent pro- and anti-angiogenic properties of MT and its benefits related to the cardiovascular system.

## MT and hypertension

Evidence has shown the close relationship between the MT levels and the incidence of cardiovascular diseases [32]. Early data showed that high blood pressure is the main cause of heart failure and MI [33]. The MT rhythmicity carefully regulates blood pressure, immune responses, homeostasis, cellular bioactivity, and respiratory chain chemical mechanisms and antioxidant defense through the above-mentioned MT receptors [34–37]. In the cardiovascular context, it has been shown that nocturnal concentrations of MT which are higher than day time levels typically decrease low blood pressure, heart rate, and increases cardiac output. The temporal increase of the MT hormone during the night could decrease high peripheral vascular resistance [38]. Previous data demonstrated less MT production, secretion, and systemic levels in patients with high blood pressure [39]. The application of β-blocking agents after myocardial infarction and other cardiovascular disorders, in turn, slows down the release of MT from pineal glands to the bloodstream [40].

It has been elucidated that the three-week course administration of MT regulated the day-night rhythm of blood pressure and reduce the possibility of ventricular hypertrophy [39]. The effects of MT in central cardiovascular are closely associated with the regulation of the suprachiasmatic nucleus and vasopressin release [41]. Tethering blood pressure via MT is done via MT1 and 2 receptors located in the tunica intima and media layers of the vascular wall and reduced circulating catecholamine levels [42]. The factor NO also plays a key role in preserving nerve and blood pressure, and exogenous administration of MT can support a significant reduction of NF-ƙB and oxidative stress by simply changing the activity of eNOS. The direct effect of MT on the endothelial intracellular calcium levels and eNOS activity has been previously shown [43]. Upon MT attachment to MT1, Gαi diminishes the intracellular level of cAMP. On the other hand, simultaneous activation of subunit Gαq activates downstream effectors such as phospholipase C which leads to the production of diacylglycerol and inositol-3-phosphate and increased intracellular calcium contents [44]. In contrast, the activation of subunit Gαq in vascular smooth muscle contributes to vasoconstriction [44]. Interestingly, the simultaneous activation of MTs receptors could lead to two completely different biological effects. For instance, MT promotes vasoconstriction by stimulating the MT1 receptor while MT interaction with the MT2 receptor enhances vasodilatation through enhances nitric oxide and soluble choline cyclase production and promotion of cGMP. It seems that activation of Gαi subunits in both MT1 and MT2 receptors, especially the MT2 receptor, in ECs overwhelms the MT-derived contraction of vascular smooth muscle cells [44]. Along with these comments, it seems that the decrease in the MT2/1 ratio could increase vessel contraction rhythms and makes the individuals susceptible to coronary artery disease. Besides, MT inhibits leukocytosis, leukocyte adhesion, and platelet aggregation via MT2 receptors [45, 46]. The interesting issue is that the reduction of the MT1 receptor occurs normally in the coronary artery endothelial cells at 2:00 am too late in the morning with a gradual increase from 13:00 to midnight [47, 48]. Previous experiments have shown that MT2 has a similar same rhythm in healthy humans and patients with coronary artery disease, whereas the bioactivity of MT2 in the coronary artery was different from the control group. Considering the nigh-day fluctuation of MT and relevant receptors either MT1 or MT2, it is logical to hypothesize that the risk of heart attack and stroke increases in susceptible people during the early morning along with a sharp drop in the levels of MT and receptors.

## Effect of MT in the promotion angiogenesis in cardiac ischemic diseases

The lack of sufficient blood supplementation and massive cardiomyocyte necrosis contributes to the uncontrolled deposition of ECM proteins such as type I collagen in the cardiac interstitial spaces [8]. In this regard, attempts have focused on the promotion of angiogenesis and blood vessel formation to the infarcted areas to reduce levels of cellular death and cardiac insufficiency. On the other hand, the promotion of angiogenesis not only diminishes the size of necrotic sites but also helps the resident cells to heal and restore the affected areas [49]. Based on released data, there is a solid inverse relationship between endogenous MT and cardiovascular diseases. In support of this statement, low MT synthesis levels correlate with an increased chance of MI, highlighting the supporting role of MT in the alleviation and prevention of cardiovascular injuries [50]. MT is touted as a unique hormone with pleiotropic effects on angiogenesis as it could mediate diverse biological effects on ECs [51] (Table 1 and Table 2).

**Table 1:** Some studies related to anti-angiogenic properties of melatonin

Study	Model	Outcome	Ref
HUVECs-human neuroblastoma cells SH-SY5Y co-culture in a paracrine and juxtacrine manner	In vitro	Paracrine VEGF↓, Tubulogenesis↓, Migration of HUVECs↓	[52]
Combined effect of ionizing radiation and melatonin on HUVECs and MCF-7	In vivo and in vitro	VEGF↓, ANG-1↓, and -2↓ , HUVECs survival↓, Migration↓, Tubulogenesis↓, Estrogen biosynthesis↓, VE-cadherin↓, Chorioallantoic membrane angiogenesis assay↓	[53]
Effect of Melatonin on VEGF-exposed HUVECs	In vitro	VEGFR-2 phosphorylation↓, PLCγ1 phosphorylation↓, pAkt/Akt ratio↓, p-eNOS/eNOS ratio↓,	[54]
Effect of melatonin on Dalton’s lymphoma angiogenesis potential	In vitro and in vivo	Dalton’s lymphoma-induced endothelial cells proliferation↓, Migration↓, Chick chorioallantoic membrane angiogenesis↓, Mouse mesentery peri-vascularization↓, TIMP3↑, VEGF↓, VEGFR↓, FGF↓	[55]
Effect of melatonin on hypoxic PC-3 prostate cancer cells	In vitro	miRNA3195↑ and miRNA374b↑, HIF-1α↓, HIF-2α↓, VEGF↓,	[56]
Effect of melatonin on tumor cells under normal condition	In vitro	phospho-STAT3↓ and CBP/p300↓, VEGFR2↓, HIF-1α↓	[57]
Effect of melatonin on hypoxic HepG2 hepatic cancer cells	In vitro	p-STAT3/STAT3↓, HIF-1α↓, VEGF↓, CBP/p300↓,Tubulogenesis↓,	[58]
Effect of melatonin on SGC-7901 gastric cancer cells	In vivo and in vitro	VEGF secretion↓, RZR/RORγ↓, SUMO-specific protease↓, HIF-1α↓	[59]
Combined effect of Melatonin and Docetaxel and Vinorelbine on angiogenesis	In vitro and in vivo	VEGF-B↓, VEGF-C↓, VEGFR-1↓, VEGFR-3↓, ANG1↓, ANG-2↓, VE-cadherin↓, Chick chorioallantoic membrane angiogenesis↓	[60]
Effect of melatonin on angiogenesis potential of MDA-MB-468 breast cancer cells	In vivo and in vitro	miR-152-3p↑, IGFR↓, VEGF↓, HIF-1α↓	[61]

*HUVECs* Human umbilical vein endothelial cells, *VEGF* Vascular endothelial growth factor, *ANG-1 and 2* Angiopoietin-1 and -2, Vascular endothelial-cadherin: VE-cadherin, *PLCγ1* Phospholipase Cγ1, Endothelial nitric oxide synthase: eNOS, *TIMP-3* Tissue inhibitor of metalloproteinase-3, *VEGR*: Vascular endothelial growth factor receptor, *FGF* Fibroblast growth factors, *HIF-1 and 2α* Hypoxia-inducible factor1α and 2α, *STAT3* Signal transducer and activator of transcription 3, *CBP* CREB-binding protein, *ROR* RAR-related orphan receptor gamma, *SUMO* Small ubiquitin-like modifier

**Table 2:** Pro-angiogenic properties of melatonin

Study	Model	Outcome	Ref
Effect of melatonin implants in the proximal metaphyseal area of rabbit right tibia	In vivo	Vascular density↑	[62]
Application of melatonin in rat corneal micropocket assay and mouse model of indomethacin-induced gastric ulcers	In vivo	MMP-2↑, VEGF↑,MMP-14↑, TIMP-2↓	[63]
Intra-dermal administration of melatonin in rat model of dermal wound healing	In vivo	iNOS↓, Cyclooxygenase-2↑, VEGF↑, Hemoxygenase-1↑, Scar formation↓,	[64]
Full-thickness incision in the model of mice	In vivo	Vascular units↑, Collagen fiber↑, hydroxyproline↑	[65]
Effect of melatonin on swine granulosa cells	In vitro	Proliferation↑, E_2_↑, P4↑, NO↓, Angiogenesis (Fibrin gel angiogenesis assay)↑	[66]
Effect of melatonin on human granulosa-lutein cell culture and Ovarian hyperstimulation syndrome	In vitro and In vivo	Follicular fluid MT2↑, P450 aromatase cytochrome↑, VEGF↑, iNOS↓	[67]
Inclusion of melatonin in collagen- poly(dialdehyde) gum acacia scaffold in rat model of excisional skin wound	In vivo	Re-epithelialization↑, Collagen deposition↑, Blood vessels formation↑	[68]
Protective effect of melatonin on EPCs after exposure to advanced glycation end product	In vitro and in vivo	EPC apoptosis↓, mitochondrial damage↓, Protective autophagy↑, tubulogenesis↑, Migration↑, AMPK/mTOR axis↑, α-SMA expressing cells↑, Collagen synthesis↑	[69]
Reno-protective effect of melatonin on early outgrowth EPCs	In vivo and In vitro	Renal function↑, TGF-β-induced apoptosis↓, VEGF secretion↑, Migration↑	[45]
Pre-treatment of adipose MSCs before administration into ischemic renal tissue	In vivo and In vitro	Cat activity↑, SOD activity↑, MSCs apoptosis↓, bFGF↑, HGF↑	[70]
Protective effect of melatonin on oxygen-glucose deprivation of brain microvascular cells	In vitro	ROS production↓, Claudin 5↑, VEGF↑, p-Akt/Akt ratio↑	[71]

*MMP* Matrix Metallopeptidase, *E*_*2*_ Estrogen, *P*_*4*_ Progesterone, *MT2* Melatonin receptor-2, *α-SMA* Alpha-smooth muscle actin, *AMPK* AMP-activated protein kinase, *mTOR* mammalian target of rapamycin, *TGF-β* Transforming growth factor beta, *SOD* (Super Oxide Dismutase, *Cat* Catalase, *HGF* Hepatocyte growth factor, *bFGF* Basic fibroblast growth factor, *EPC(s)* Endothelial progenitor cells

It has been shown that MT increases the release of pro-angiogenic factors from immune cells, stem cells, and ECs. Once cardiac infarction is initiated, infiltrated immune cells were recruited to the site of injury to remove dead cells and to promote heart tissue healing. The simultaneous application of stem cells plus MT could increase the pro-angiogenic paracrine activity of stem cells rather than cell differentiation via RAP1/NF-ƙB signaling pathway [72]. The production of factors related to the inhibition of apoptotic and stimulation of angiogenesis is crucial for the successful injury-repair in the infarcted area. For example, MT or Serotonin is a stimulator for megakaryopoiesis and proplatelet formation [73]. MT-induced platelet-derived growth factor is a putative anti-apoptotic factor with the capability to stimulate angiogenesis in immune cells or stem cells beneficial for heart repair [74, 75]. Commensurate with these comments, it is valuable to include these contents for more insights into the understanding of multiple facets of MT in the treatment of cardiovascular injuries. Nevertheless, previous experiments did not answer the question of whether MT is beneficial or detrimental to angiogenesis. It seems that MT regulates the angiogenic response in a dose- and context-dependent manner. The pharmacological concentration of MT typically inhibits the angiogenesis rate via suppressing VEGF and different intracellular signaling pathways in the tumor niche [1]. The inhibition of angiogenesis in the presence of MT is associated with the regulation of angiogenesis-related genes such as erythropoietin, NOS, and VEGF by suppressing HIF-1α coincided with the prohibition of STAT3 [39]. Besides, some authorities have shown that the stimulation of VEGFR-2 by VEGF results in an increased ROS production but the close relationship between VEGFR-ROS interaction is still missing. It has been hypothesized that the stability of HIF-α was increased in the presence of ROS [1]. Due to the direct and indirect activity of MT on ROS metabolism, it is mighty to mention that MT could regulate the function of angiogenesis in different contexts, supporting the multi-organ effects of MT [1].

The distribution and balance of MT receptors (MT1 and MT2) are tissue-dependent and it won't be unreasonable if we imagine that MT could exhibit a different angiogenic potential at the same doses [76]. Even, MT at physiological doses could show different angiogenic potential regarding the participation of specific regulatory effectors and the nature of distinct tissue structures [6]. MT displays a striking impact on the expression of certain genes related to oncogenesis. For instance, MT reduces the expression of c-Myc via increasing Bridging Integrator 1 in hepatocellular carcinoma [77]. Bridging Integrator 1 is a factor required for cardiac muscle development and inhibition of apoptosis during cardiac injury. Given a large number of studies on the inhibitory effects of MT exist in the field of cancer biology, we focus on how MT supports angiogenesis and blood nourishment in the field of cardiovascular disease. Except for normal conditions, the promotion of angiogenesis coincides with the inflammatory response and pathological changes. In these circumstances, the levels of ROS and pro-angiogenesis factors are elevated proportionally. For example, hypoxia and ischemic changes activate the HIF-1α in response to the accumulation of ROS [78]. A great body of documents showed that both VEGF and ROS play an inevitable role in vascular pathophysiology [1].

In the hypoxic and ischemic areas, necrotic tissues are developed in the myocardium, termed also the infarct zone. During ischemic injury of cardiac reperfusion, excessive production of ROS occurs coincided with the elevation of intracellular calcium and pro-inflammatory cytokines, leading to the reduction of ATP, irreversible protein, fat, and DNA oxidation. These features promote the initiation of apoptotic changes in the cardiomyocytes [79]. Calling attention, the component of the redox signaling pathway plays an inevitable role in the therapeutic effects of MT. For instance, it was mentioned that MT can stimulate the transcription of AP-1 and Nrf2 and thereby intensify the antioxidant mechanisms. MT is potent to decrease the systemic level of adrenaline, regulate the intracellular level of calcium required for the dilatation of myocardial arteries. These features contribute to minimal cellular mitochondrial deficiency due to the increase of Bcl-2 [80, 81].

SIRT1 is a NAD-dependent histone that activates the synthesis of antioxidants such as Mn-SOD and Cat, to enhance cell resistance against insulting conditions and oxidative stress. MT, a potent SIRT1 regulator, can control the expansion and promotion of different cell deaths such as apoptosis and autophagy. In this regard, studies have shown the role of SIRT1 in the improvement of cardiac function impairment due to transplantation and septicemia [82, 83].

Even in light of previous experiments, the impact of MT on angiogenesis remains controversial and sometimes there are controversies. To gain insight into the mechanisms by which MT increases the angiogenesis potential, researchers have conducted numerous experiments. In a study conducted by Zhu and co-workers, they pre-treated adipose-derived mesenchymal stem cells with 5 μM MT for 24 hours before transplantation into the rat model of myocardial infarction [84]. According to data, MT enhanced both pro-angiogenic and mitogenic factors activity of these cells by increasing the synthesis factors such as like IGF-1, bFGF, HGF, EGF while the contents of VEGF and G-CSF were unchanged [84]. It is thought that this prevailing angiogenesis response is related to paracrine activity after the treatment of stem cells with MT [85]. Similar results also were obtained by other researchers who showed that MT-pretreated MSC transplantation into ischemia stroke induced by middle cerebral artery occlusion decreased brain infarction via the synthesis of VEGF through the Erk1/2 pathway [86]. Lee and colleges found that treatment of MSCs with MT promoted mitochondrial activity via activation of PGC-1α in hind limb ischemia. They found that MT increased secretion of angiogenic cytokines and migration of MSCs after grafting into the ischemic area [87]. Mias et al. found enhanced CD31 and α-SMA positive cells after intra-parenchymal injection of MT-treated MSCS into a rat model of acute renal failure [70]. It seems that MT, not only, increases the angiogenic potential of MSCs, but also, makes these cells resistant to the ischemic stress via the promotion of cell survival mediated in part by MT1, MT2 receptors [70]. Most of the functional studies have focused on the differentiation potency of stem cells at transplant sites. But the truth is that accumulation of endogenous ROS and active inflammatory cytokines, mainly TNF-α, could contribute to early stemness loss and senescence. It was shown that MT reversed negative inflammation effects in bone marrow MSCs via MT1, MT2 receptors, and up-regulation of YAP [88]. The factor YAP could regulate the function of STAT3 and RUNX2 and β-catenin which are involved in cell fate decisions [88]. It has been speculated that the positive effect of MT on angiogenesis is, in part, due to increased paracrine activity [89]. MT exhibited a stimulatory action on the expression of S-100 protein in adrenal gland sustentacular cells and telocytes coincided with the increase of sinusoidal vessel dendritic cells. According to data obtained from the recent study conducted by Hussein and co-workers, MT increased the number of vimentin-positive stem cells in the adrenal gland, and close collaboration of MT-exposed stem cells with telocytes expanded vascular network. They also reported a large number of secretory vesicles in the cytoplasm of telocytes after treatment with the MT [89]. To date, numerous studies confirmed the protective role of MT on myocardial I/R injury in different animal models and clinical trials [90, 91]. Another explanation of the stimulatory effect of MT angiogenesis is related to the quantitative and qualitative changes in the content of stem cell exosomes [92]. Alzahrani declared that transplantation of exosomes from MSCs pre-treated with MT could improve pathological changes in a rat model of renal I/R [92]. He concluded that the exosomes of MT-treated MSCs harbor a large amount of VEGF factor which could promote neovascularization in the renal tissue after I/R. Similar advantages were observed when chronic kidney disease-derived MSCs were exposed to MT. It was shown that MSCs from the mouse with chronic kidney disease were susceptible to senesces and oxidative stress [79]. MT treatment protects these cells from excessive senescence via activation of mitochondrial function and up-regulation of the cellular prion protein. The paracrine angiogenic potential of MT-treated MSCs was also enhanced by enhanced VEGF, FGF, and HGF secretion [79]. These data showed that MT can interfere with the interaction with cell-surface glycoprotein, namely cellular prion protein, and mitochondrial function, and angiogenic potential. However, the close relationship between cell-surface glycoprotein and mitochondrial activity triggering pro-angiogenesis synthesis has yet to be elucidated. Another aspect relevant to MT-derived mitochondrial protection is associated with the activation of SIRT1, a histone deacetylase, in the rat model of myocardial ischemic–reperfusion [82]. It seems that MT yields highly effective protection against myocardial ischemic–reperfusion injury via the stimulation of SIRT1. The promotion of SIRT1 could suppress the activation of pro-apoptotic factors such as Bax and maintain the normal activity mitochondria in ischemic areas [82]. Besides, MT prevents mitochondrial membrane permeability and cytochrome C leakage to the cytosol by engaging mitochondrial MT_1_/Gai protein and modulation of AMPK which in turn regulates the mitochondria health and cardiomyocytes' mitotic activity [93]. At the time of the myocardial infarction and/or ischemic changes, various types of inflammatory mediators and cytokines such as IL-1β are produced which alone activates Toll-like receptor-4 receptors and TNF-α. Studies have also reported a positive effect of melatonin on the reduction of caspase levels and IL-1β [29].

## Conclusion

Owing to the pleiotropic and multi-organ effects of MT, most of the previously published data highlighted the role of MT in the inhibition of cancer angiogenesis. However, the existence of anti-angiogenic properties does not mean that MT could not exert a pro-angiogenic outcome in a different milieu. Regarding context-specific angiogenesis controlling mechanisms, it seems that MT is a smart molecule and could accelerate regeneration and healing procedures by induction of angiogenesis (Fig. 3) while an inhibited angiogenesis response has been shown in the cancer microenvironment. Therefore, a critical question to be answered is how MT acts as a pro- and anti-angiogenesis agent, and attempts should be focused on the finding of relative pro- and anti-angiogenic concentrations.

## Data Availability

None applicable.

## Abbreviations

AP-1: Activator protein-1
NAD: Adenosine dinucleotide nicotinic amide
AMPK: AMP-activated protein kinase
bFGF: Basic fibroblast growth factor
Cat: Catalase
ECs: Endothelial cells
EGF: Epidermal growth factor
ECM: Extracellular matrix
GPx: Glutathione peroxidase
GR: Glutathione reductase
G-CSF: Granulocyte colony-stimulating factor
HGF: Hepatocyte growth factor
HIF-1α: Hypoxia inducer factor-1α
IGF-1: Insulin-like growth factor 1
IL-1β: Interleukin-1β
I/R: Ischemia-reperfusion
MT: Melatonin
MI: Myocardial infarction
NO: Nitric oxide
eNOS: NO synthetase
NF-ƙB: Nuclear factor kappa
PGC-1α: Peroxisome proliferator-activated receptor-gamma coactivator-1 alpha
ROS: Reactive oxygen species
SIRT1: Sirtuin1
SDF-1α: Stromal cell-derived factor-1
SOD: Superoxide dismutases
TGF-β 1 and 2: Transforming Growth Factor
VEGF: Vascular endothelial growth factor
